# The role of ^90^Y-radioembolization in downstaging primary and secondary hepatic malignancies: a systematic review

**DOI:** 10.1007/s40336-016-0172-0

**Published:** 2016-04-23

**Authors:** M. N. G. J. A. Braat, M. Samim, M. A. A. J. van den Bosch, M. G. E. H. Lam

**Affiliations:** 1 Department of Radiology and Nuclear Medicine, University Medical Center Utrecht, Heidelberglaan 100, 3584 CX Utrecht, The Netherlands; 2 Department of Surgery, University Medical Center Utrecht, Utrecht, The Netherlands

**Keywords:** Radioembolization, Downstaging, Bridge to transplant, Future liver remnant

## Abstract

Radioembolization (RE) is an emerging treatment strategy for patients with primary hepatic malignancies and metastatic liver disease. Though RE is primarily performed in the palliative setting, a shift toward the curative setting is seen. Currently, hepatic resection and in selected cases liver transplantation are the only curative options for patients with a hepatic malignancy. Unfortunately, at diagnosis most patients are not eligible for liver surgery due to the imbalance between the necessary liver resection and the remaining liver remnant. However, in borderline resectable cases, tumor volume reduction and/or increasing the future liver remnant can lead to a resectable situation. The combination of selective tumor treatment, the induction of hypertrophy of untreated liver segments, and its favourable toxicity profile make RE an appealing strategy for downstaging. The present review discusses the possibilities for RE in the preoperative setting as a downstaging tool or as a bridge to liver transplantation.

## Introduction

The incidence of both primary and secondary hepatic malignancies is continuously increasing worldwide [1–3]. At the same time, treatment strategies have changed considerably in the last two decades and continue to evolve. Though treatment strategies vary substantially between primary [most common types: hepatocellular carcinoma (HCC) and intrahepatic cholangiocarcinoma (ICC)] and secondary liver malignancies and their individual subtypes, less than 30 % can be curatively resected at diagnosis [4–7]. However, the number of patients with resectable disease can be increased, if the individual tumor load is decreased (i.e. downstaging) [8].

Metastatic liver disease is far more common than primary hepatic malignancy, with colorectal malignancy being the most common tumor type. Hepatic resection is considered the only potentially curative option for colorectal cancer liver metastasis (CRLM) with 5- and 10-year survival rates approaching 60 and 25 %, respectively [7, 9, 10]. Ongoing improvements in chemotherapeutic regimens, the addition of monoclonal antibodies and the more liberal attitude towards hepatic resections, have led to a significant increase in the number of hepatic resections [4, 5, 7, 8, 10, 11]. In the series of Adam et al. 13 % of patients with initial unresectable CRLM underwent liver surgery after downstaging with a 5-year disease-free survival (DFS) of 22 % and a 5-year overall survival (OS) of 33 % [5].

The number of hepatic resections is even further increasing since metastases of other primaries are progressively treated with surgery, such as breast carcinoma, melanoma, GIST and neuroendocrine tumors [12, 13]. For example, the reported 5-year survival rates in carefully selected populations are 21–60 % for breast carcinoma and up to 60–80 % for neuroendocrine tumors.

Contrary to metastatic liver disease (apart from selected cases with metastases from neuroendocrine tumors), liver transplantation (LTX) is part of the standard curative treatment arsenal in patients with limited HCC and in rare cases of ICC [14–17]. HCC nearly always develops in patients with known liver disease (mostly cirrhosis due to HBV or HCV) and thus often compromised liver function [14, 18]. LTX has the advantage of treating the underlying liver disease and associated future “de novo” HCC risk, leading to better overall and recurrence-free survival (RFS) than hepatic resection. Patient selection for LTX is strict and generally based on the Milan criteria (a solitary lesion of <5 cm in diameter or up to 3 lesions all <3 cm in diameter and no macroscopic vascular invasion or extrahepatic metastasis) [19]. Adherence to these criteria has resulted in 5-year survival rates of >70 %, slightly worse than survival rates after LTX for non-tumorous conditions (3-year survival of 71 vs 84 % [20]) [14, 15, 19, 21, 22]. Paucity of donor organs has made resection a reasonable alternative for LTX in selected cases (patients with a solitary HCC <5 cm and a Child Pugh A score without portal hypertension) [23, 24]. Furthermore, in patients with an HCC <3 cm ablative techniques, such as radiofrequency ablation, are also a curative option [24].

Negative resection margins are an important prognostic factor for survival in both primary and secondary hepatic malignancies [10, 25, 26]. Rees et al. reported in a cohort of 929 CRLM patients a 5-year survival of 18 % in R1 resections compared to 40 % in R0 resections [10]. Similarly, Spolverato et al. reported an incrementally worsening RFS and OS with decreasing margin width in ICC patients [26]. Several factors have been associated with positive resection margins, such as multiple lesions, bilobar disease, tumor size, major liver resections, vascular reconstruction/invasion and caudate lobe resections [25–27]. Downstaging strategies aim to reduce abovementioned factors, thus hoping to improve OS. Fortunately, preoperative imaging can assess the presence of these factors adequately, facilitating the decisions on downstaging and surgical treatments.

Radioembolization (RE) is an emerging treatment strategy for patients with hepatic malignancy. Hepatic tumors are targeted by the injection of radio-active microspheres into the hepatic or tumor supplying arteries, resulting in selective radiation of these tumors. Currently microspheres with varying radio-isotopes are being tested in clinical trials, however only two types of microspheres are commercially available, both embedded with yttrium-90 (^90^Y): glass spheres (Theraspheres^®^, BTG International, London, England) and resin spheres (SIR-Spheres^®^, Sirtex Medical, North Sydney, Australia). Until now RE is primarily performed as a salvage treatment, yet the qualities of RE (such as selective tumor targeting, the lack of heat-sink effect near the great vessels and the induction of hypertrophy in non-embolized lobes) make its use in the curative setting, as an adjunct to surgery for example, appealing.

In this systematic review, we will discuss the potential of RE in downstaging and as a bridge to liver transplantation.

## Downstaging or bridging to LTX in general

Pre-transplant locoregional liver therapies are mainly focused on preventing drop-out from the LTX waiting list in patients with an HCC within the Milan criteria (i.e. bridge to LTX) or on downstaging to meet the Milan criteria (instead of enabling hepatic resection, as is the case in ICC and metastatic disease) [23, 24, 28]. Several studies have shown that drop-out due to tumor progression is rare in the first 3 months after enlisting, but drop-out numbers increase with longer waiting times (up to 57 % after 12 months) and also with increasing lesion size and number [18, 22, 28, 29]. Pre-transplant locoregional liver therapies have shown to reduce the drop-out rates [20, 29, 30] and increase long-term post-transplant patient and graft survival [20]. Currently up to 65 % of patients within the Milan criteria receive bridging therapies, primarily in the form of trans-arterial chemoembolization (TACE) or radiofrequency ablation (RFA) [18, 20, 22, 24]). TACE seems preferable in HCC lesions >3 cm or multifocal HCC, while RFA seems most promising as a bridging strategy in HCC lesions <3 cm [24, 28].

Several studies have addressed the potential of TACE as a downstaging treatment strategy [29–35]. Yao et al. reported successful downstaging to the Milan criteria in 70 %, with a 4-year survival of 92 % [29]. Others reported similar transplantation rates, tumor recurrence rates and survival rates compared to patients within the Milan criteria [30, 33–35]. Tumor recurrence rates were however influenced by tumor progression in the waiting time [33, 35]. Therefore, a waiting time of 3 months from the enlisting for LTX is recommended in order to select HCC’s with less aggressive biological behaviour and prevent recurrences [24].

Most institutions currently reserve LTX for patients with HCC within the Milan criteria, as validated by the United Network for Organ Sharing (UNOS). However, some centers apply expanded criteria, such as the UCSF criteria or up-to-7 criteria.

## Search strategy

A PubMed literature search was performed on 11 November 2015 to identify all articles related to the use of RE in downstaging of liver malignancy or as a bridge to LTX. Search terms used to identify these articles were ‘radioembolization’, ‘downstaging’, ‘hepatectomy’, ‘bridge to transplant’, ‘liver remnant’ and their synonyms. This search yielded 148 articles. The following exclusion criteria were applied: animal studies, reviews, metaananalyses, conference abstracts, consensus statements and protocol publications, and languages other than English or German. After application of these exclusion criteria 42 articles were screened on full-text. Another 13 articles were excluded [no data on the downstaging success rate (*n* = 11) or only data after combined multimodality liver therapy (*n* = 3)]. The remaining 21 original studies and 7 case reports were included in this review [6, 36–60]. Cross-referencing of their references yielded 15 relevant additional publications [61–75].

## Downstaging with RE

Several prospective and retrospective studies in patients with intermediate HCC (not eligible for resection or LTX) have shown multiple advantages of RE over TACE. RE was associated with a longer overall survival, longer time to progression, faster time to radiological response, shorter hospitalization, less postembolization syndrome and a smaller number of treatment sessions, while having a similar toxicity profile [51, 64, 69, 76]. In case of RE with lobar delivery, another possible advantage is treatment of non-detected HCC nodules, as these are reported in 36 % of the liver explants [32]. These advantages make RE an attractive option for downstaging and as a bridging therapy.

A few small studies and case reports have reported on RE in this context (Table 1) [6, 36, 40, 45, 47–51, 57, 58, 73]. The largest study compared TACE and RE in a non-randomized cohort study of 86 patients with UNOS stage T3 HCC [51]. Downstaging to UNOS T2 HCC occurred in 58 % after RE and in 31 % after TACE (*p* = 0.02). This resulted in LTX in 21 % after RE and 26 % after TACE, whereas 42 and 23 %, respectively were downstaged to RFA. OS was better after RE than after TACE (3-year survival of 59 vs 19 %; *p* = 0.008), as was 1-year RFS after LTX (89 vs 73 %). Others report similar downstaging success rates of 29–50 % [6, 40, 57]. Kulik et al. performed a pilot study to assess the benefit of adding Sorafenib to RE in patients awaiting LTX [48]. Tumor size reduction was comparable in both groups, but biliary complications and LTX rejection were only encountered in the RE + Sorafenib group. Based on the limited available evidence, applying RE as a tool for downstaging or bridging to LTX in HCC seems feasible. However, RCTs are mandatory to further investigate its potential.

**Table 1:** Overview of response rates and downstaging success rates with ^90^Y-RE in patients with HCC

Author	Year	*N*	mRECIST (%)		WHO (%)		EASL (%)		Downstaging success rate	Median time to response/downstaging	Resection or RFA	LTX
			CR	PR	CR	PR	CR	PR	%	Months (range)	%^e^	%^e^
Kulik^a^ [49]	2006	34	–	–	–	50	–	–	67	4 (1.9–16.3)	34	23
Heckman [43]	2008	16	–	–	–	–	–	–	13	–	–	100^f^
Lewandowski^a^ [51]	2009	43	–	–	0	61	47	39	58	3.1 (1.8–8.7)	42	21
Ibrahim^a^ [45]	2012	8	–	–	13	63	37	50	50	–	–	37
Iñarrairaegui [6]	2012	21	–	–	–	–	–	–	29	–	19	10
Tohme [57]	2013	20	37	19	–	–	–	–	33	–	–	100^f^
Donahue^b^ [108]	2013	12	–	–	0	50	25	42	8	(1.4–11.3)	–	50
Vouche [75]	2014	102	47	39	–	–	–	–	–	–	–	32
Ettorre^c^ [40]	2014	22	–	–	–	–	–	–	50	–	5	45
Kulik^d^ [48]	2014	20	–	–	–	–	–	–	–	–	5	85
Abdelfattah [36]	2015	9	–	–	–	–	–	–	100	–	–	100^f^

^a^Overlapping patientpopulations

^b^Prospective study on ^90^Y-RE in patients with a transjugular intrahepatic portosystemic shunt (TIPS)

^c^Correspondence to editor

^d^Prospective randomized pilot study comparing ^90^Y-RE + Sorafenib with ^90^Y-RE alone

^e^Percentage of the total population

^f^100 % LTX is inherent to retrospective patient study design

In case of intrahepatic cholangiocarcinoma (ICC), downstaging is less often reported. Apart from few case reports, two institutions have reported their experiences with ^90^Y-RE in downstaging (Table 2) [53, 63, 65, 67, 70]. Rayar et al. reported successful downstaging to resection with RE and chemotherapy in eight patients with initially unresectable ICC [70]. R0 resections were achieved in all patients, with a median of six resected segments [70]. Mouli et al. reported successful downstaging to resection in 5/46 patients with unresectable ICC and successful LTX in one patient [53].

**Table 2:** Overview of response rates and downstaging success rates with ^90^Y-RE in patients with ICC

Author^a^	Year	*N*	RECIST (%)		WHO (%)		EASL (%)		Downstaging success rate	Median time to response/downstaging^b^	Resection	LTX
			CR	PR	CR	PR	CR	PR	%	Months (range)	%^c^	%^c^
Ibrahim [67]	2008	24	0	27	–	–	9	77	8	–	4	4
Mouli [53]	2013	46	–	–	0	25	9	64	13	3.6	11	2
Rayar [70]	2015	10	–	–	–	–	–	–	80	7.6 (3.4–16.7)	–	0
Edeline [63]	2015	24	0	25	–	–	–	–	46	–	46	0

^a^The patientpopulations of Ibrahim and Mouli (partially) overlap, as well as the populations of Rayar and Edeline

^b^Rayar et al.: median time from start chemotherapy to resection in a study on chemotherapy and ^90^Y-RE as first-line treatment for ICC

^c^Percentage of the total population

Similar to HCC and ICC, the evidence for downstaging of metastatic liver disease with RE is limited, with a few case reports/case series (<5 patients) and one small clinical study (Table 3) [37, 38, 42, 46, 52, 58, 59, 71]. Justinger et al. reported on 13 CRLM patients with marginally resectable disease, who were treated with resin microspheres for intended downstaging [46]. Hepatic resection was performed in 11/13 patients after a median of 57 days (range 39–153) following RE; combined with ALPPS (associating liver partition and portal vein ligation for staged hepatectomy) in 7/11 and with PVE in 1/11.

**Table 3:** Overview of response rates and downstaging success rates with ^90^Y-RE in patients with metastatic disease

Author	Year	*N*	RECIST (%)		WHO (%)		EASL (%)		Downstaging success rate	Median time to response/downstaging	Resection	LTX
			*CR*	PR	CR	PR	CR	PR	%	Months (range)	%^d^	%^d^
Whitney [59]	2011	44	–	–^a^	–	–	–	–	9	–	9	0
Vouche [58]	2013	8	–	–	–	–	–	–	13	–	13	0
Moir^b^ [52]	2015	44	0	10	–	–	–	–	16	4.0 (2.3–10.9)	16	0
Justinger [46]	2015	13	–	–	–	–	–	–	85	1.8 (1.3–4.9)	85	0
Henry^c^ [44]	2015	9	–	–	–	–	–	–	29	3.8 (1.8–8.3)	100	0

^a^All surgical candidates (*n* = 4) had PR according to RECIST

^b^Mixed population (CRLM 14, 8 HCC, 5 NET, 4 other); 4/14 CRLM underwent surgery

^c^Retrospective series with a mixed population of secondary liver malignancies (CRLM 4, 3 NET, 1 gastrointestinal stromal tumor and 1 cervical carcinoma)

^d^Percentage of the total population

The role of RE in downstaging prior to ablation therapy has also been investigated [49, 66]. Hoffman et al. reported the results of RFA in patients with extensive hepatic metastases three months after RE. Downstaging to a tumor size suitable for RFA (<3 cm) was achieved in 12 % of patients (5/44) [66]. Kulik et al. reported successful downstaging of HCC lesions (to <3 cm) by means of RE in 32 % of patients (11/34) [49].

## Future liver remnant (FLR)

Liver failure after hepatectomy, i.e. an insufficient liver remnant, currently is the major cause of postoperative mortality, especially after major resections and in patients with liver parenchymal disease (mainly cirrhosis) [77, 78]. In a large series by Cescon et al. (*n* = 1500) the incidence of transient liver failure was 4.1 %, while liver failure related mortality occurred in 1.7 % [77].

On the other hand, lethal liver failure after RE, i.e. lethal radioembolization-induced liver disease (REILD), occurs in up to 5 % of patients in large series with CTCAE grade 3 bilirubin toxicities in up to 19 % [79–81]. Consequently, RE is likely to result in some compromise of the FLR function. Yet, in the abovementioned studies, complications after RE downstaging and surgery were scarcely reported [6, 40, 46, 48, 57, 59]. However, Henry et al. reported a considerably higher 30-day mortality in patients who were treated with RE before resection than those who were not (33 vs 3 %) [44]. Liver failure related mortality after RE and resection was reported in one case [70]. Others report complication rates comparable to hepatic surgery without prior RE [46, 48, 57].

In the current guidelines, the thresholds for an adequate FLR are based on volumetric measurements. A FLR is considered sufficient if it comprises >20 % [of the initial total liver volume (TLV)] in non-exposed livers, >30 % after heavy chemotherapeutic pretreatment and >40 % in cirrhotic livers [4, 82]. Extensive resections are often in conflict with an adequate FLR. Portal vein embolization (PVE), portal vein ligation (PVL) and in situ liver splitting techniques (e.g. ALPPS) are commonly applied to overcome this problem and increase the FLR [83]. A FLR increase of 44–69 % is reported within 3–8 weeks after PVE of the right liver lobe, with an increase of the relative FLR volume [=FLR volume/(total liver volume − tumor volume)] up to 47 % [84–86]. After 12 months FLR increases of 83 % are reported [87]. Remarkably, FLR increase is more pronounced in small FLR and, as can be expected, less pronounced in cirrhotic livers [83, 85, 88]. The downside of PVE/PVL is the induction of tumor growth in the embolized and non-embolized lobes [84, 86, 88, 89], thus counteracting the downstaging strategy. This tumor increase can be up to 21 % in the treated lobe [88]. Furthermore, a considerable number of patients will become definitely ineligible for surgery due to the development of new lesions in the designated FLR post-PVE (up to 9 %) [84, 86, 89].

**Fig. 1:**
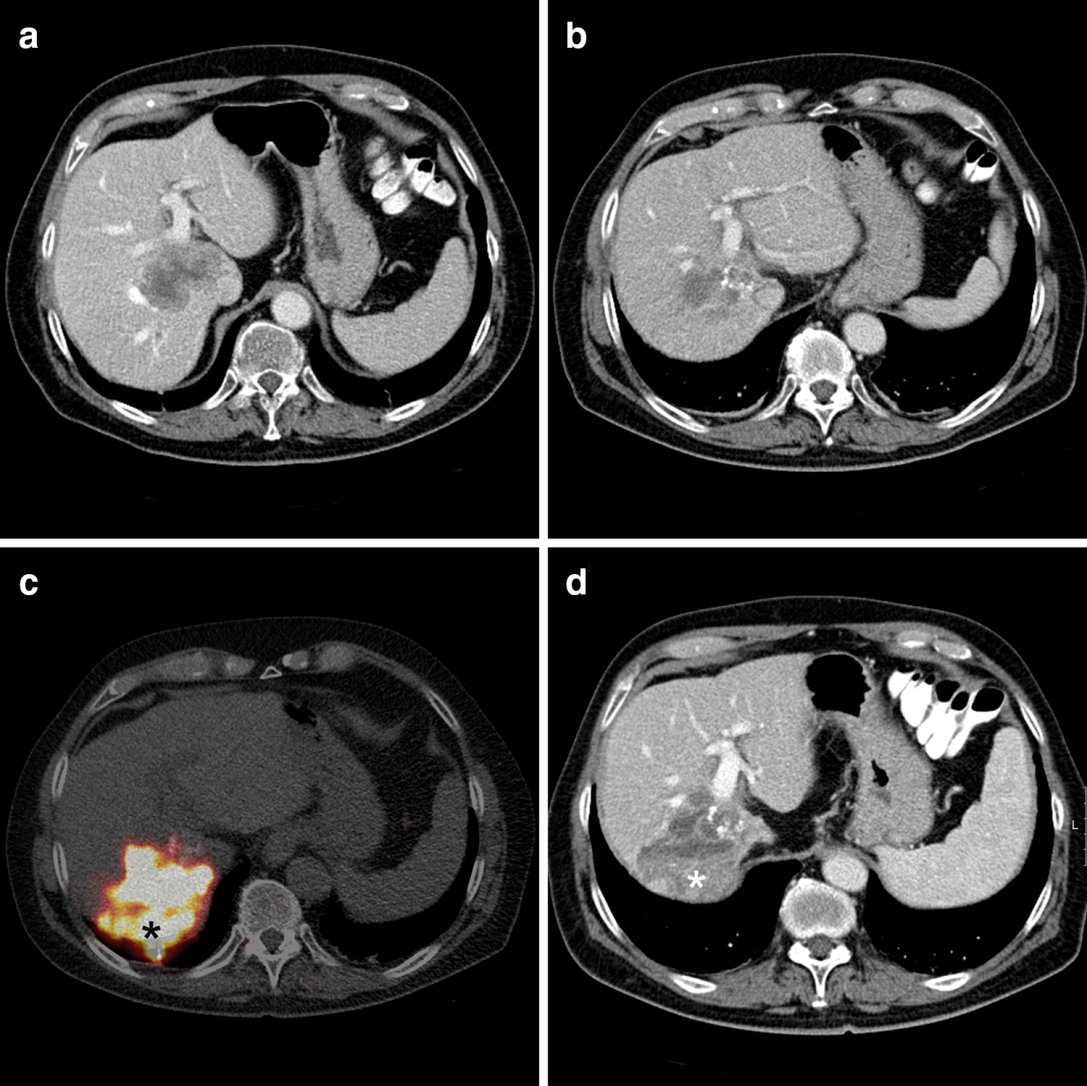
Induction of hypertrophy after 2 RE-treatments in a patient with CRLM. **a** CT scan prior to the first treatment with a CRLM located centrally in the right hemiliver, also involving the caudate lobe. **b** Three months after a whole liver treatment a decrease in the lesion size is seen. Segment 2–3 have hypertrophied (degree of hypertrophy: 16 %). **c** ^90^Y-PET/CT after a second selective treatment with glass microspheres (8 months after the first RE treatment): an intense accumulation of ^90^Y is seen in the lesion (*). **d** CT scan 2 months after the second treatment. The lesion in the right hemiliver has further decreased in size. A wedge-shaped hypodense area surrounds the lesion, consistent with radiation changes of the surrounding parenchyma (corresponding to the normal parenchyma with intense ^90^Y uptake on c (*). The hypertrophy of segment 2–3 has increased (degree of hypertrophy: 25 %). Also, segment 4 has hypertrophied (degree of hypertrophy: 20 %)

Hypertrophy of the untreated lobe(s) is a well-known by-effect of RE [6, 46, 58, 68, 72]. Hypertrophy after RE develops at a slower pace and to a lesser extent than in case of PVE/PVL with FLR increases of ca. 23 % within 1–3 months after treatment (Table 4). Even so, hypertrophy continues with FLR increases of 31–34 and 40–45 % after 6 and 12 months, respectively [56, 58]. Also, contrary to PVE, RE does have a coinciding anti-tumoral effect in the treated lobe [41]. This will allow for a longer interval to surgery and thus time to develop hypertrophy (Fig. 1). The inherent benefit of the prolonged waiting period is the possibility to assess previously undetected contralateral metastases or synchronous HCC, since the occurrence of tumor progression in the non-treated lobe after RE is comparable to PVE (Table 4).

**Table 4:** Induction of hypertrophy after ^90^Y-RE

Author	Year	Patients	Follow up period	Volume measurement	Degree of hypertrophy contralateral lobe^a^	Degree of atrophy treated lobe^a^	Response assessment	Response treated lobe	Response non-treated lobe
Jakobs [68]	2008	32	139 days	CT/MRI	21 %	9 %		–	–
Gaba [65]	2009	20	3 months	CT/MRI	40 %	52 %	EASL	CR 40 % PR 50 % SD 10 %	NA
Ahmadzadehfar [37]	2013	24	44–66 days	MRI	47 %	8 %	PERCIST and RECIST	CR 8 % PR 74 % SD 8 % PD 8 %	50 % PD
Edeline [39]	2013	34	3 months	CT	29 %	23 %^b^	mRECIST	CR 30 % PR 33 % SD 30 % PD 7 %	NA
Vouche [58]	2013	83	1 month 1–3 months 3–6 months >9 months	CT/MRI	7 % 24 % 35 % 45 %	2 % 4 % 21 % 32 %		–	20 % new lesions
Garlipp [41]	2014	35 141^b^	46 days 33 days^b^	MRI	29 % 62 %^b^	8 % 12 %^b^	RECIST	CR 4 % PR 19 % SD 73 % PD 4 %	8 % new lesions 56 % lesion growth
Teo [73]	2014	17	5,7 months	CT	34 %	22 %	RECIST	CR 12 % PR 29 % SD 35 % PD 24 %	NA
Theysohn [56]	2014	45	1 month 3 months 6 months 9 months 12 months	CT	7 % 23 % 31 % 36 % 40 %	5 % 23 % 34 % 41 % 45 %		–	–

*NA* not applicable

^a^Degree of hypertrophy: (volume non-treated lobe posttreatment−volume non-treated lobe at baseline)/volume non-treated lobe at baseline. Degree of atrophy is calculated likewise. Median volume changes are reported in the study of Gaba and Vouche. The others report mean volume changes

^b^Matched pair analysis of RE vs PVE; PVE data are marked

Theoretically, the degree of hypertrophy induction might vary with regard to the used microsphere. The lower activity per microsphere of resin spheres (50 vs 2500 Bq/sphere in glass microspheres) results in a higher amount of injected particles (i.e. embolic load), suggestive of more flow redirection. Edeline et al. compared both types of microspheres without finding a significant difference in the maximal degree of hypertrophy, though the number of treatments with resin microspheres was very low (*n* = 4) [39]. In the light of downstaging with RE, further investigation of these differences is required.

## Assessment of the FLR function

Liver function encompasses multiple subfunctions, such as synthetic, excretory and detoxicifying functions. Several tests are used to assess total liver or FLR–function, though none are able to weigh the entire spectrum of different liver functions. Some tests are based on biochemical and clinical findings, such as the Child-Pugh score and MELD score. Others are based on the liver uptake of one substance, for example the indocyanine green clearance (ICG) test and the galactose elimination capacity.

Currently, non-invasive preoperative assessment of liver function, using nuclear imaging techniques (hepatobiliary scintigraphy) is gaining ground. Two liver-specific radiopharmaceuticals are commonly used: ^99m^Tc- galactosylneoglycoalbumin (^99m^Tc-GSA) (not available in Europe and the United States) and ^99m^Tc-iminodiacetic acid (^99m^Tc-IDA) [90, 91]. ^99m^Tc-GSA is endocytosed and degraded by hepatocytes after binding to the asiologlycoprotein receptor. ^99m^Tc-IDA is processed by hepatocytes by the same organic anion-transporting polypeptides (OATP 1B1 and 1B3) and multidrug resistance protein (MPR2) as bilirubin and ICG [92, 93]. Thus, hepatic uptake of IDA analogs is influenced by hyperbilirubinemia, while ^99m^Tc-GSA uptake generally is not [90, 94]. ^99m^Tc mebrofenin is the most used IDA analog, because it has the strongest resistance to displacement by bilirubin and the highest hepatic extraction fraction.

Several hepatobiliary scintigraphy (HBS) studies have shown that there is a decreased hepatic uptake in patients with parenchymal disease and that there is little to no correlation between hepatic uptake and liver volume (especially in compromised livers) [78, 82, 93–96]. Additionally, as reported by Bennink et al., a strong association exists between the preoperatively determined FLR function and the actual liver remnant function 1 day after surgery as measured by HBS (*r* = 0.95, *p* < 0.001) [95]. Also, in a study by Dinant et al. HBS was reported to be more accurate in the prediction of postoperative liver failure than CT volumetry [78]. Their results indicated that a safe resection was possible in patients with a FLR uptake of >2.5 %/min/m^2^ of body surface area (with a 3 % chance of liver failure development above this uptake value).

However, in current practice FLR sufficiency is often still based on volumetric measurements; even when (the extent of) underlying parenchymal disease is not known preoperatively. Apart from the inadequate quantification of the function of the FLR parenchyma by volumetry, regional differences are not accounted for. Inhomogeneous liver function distribution is quite common, especially in cirrhotic livers, in case of a hilar cholangiocarcinoma and after PVE [82, 91]. In contrast to CT and MRI volumetry, HBS can image regional and segmental differences in liver function, especially when combined with SPECT/CT [82, 97]. Another advantage of HBS SPECT/CT is the better delineation of the separate segments and thus the FLR, when compared to the planar HBS 2-dimensional images [82]. Interestingly, few authors have reported on HBS after PVE [94, 97, 98], with consistent results of a larger increase in FLR function than in FLR volume in both normal and compromised livers. This faster functional increase argues for a shorter interval between PVE (or RE) and surgery, even when volumetric hypertrophy is not yet up to par.

Up to date, only one report of HBS imaging after RE has been published. Bennink et al. reported on 2 cases with multifocal HCC undergoing HBS (with ^99m^Tc-mebrofenin) both prior to and 6 weeks after RE [61]. After RE both patients had a reduced total liver function [reduced body surface area corrected ^99m^Tc mebrofenin uptake rate (cMUR)] due to an uptake decrease in the treated lobe(s). One patient underwent two whole liver treatments in 6 months, resulting in a reduction in cMUR_total liver_ from 7.4 to 6.1 %/min after the first treatment and from 4.8 to 2.2 %/min after the second treatment. This patient was thereafter diagnosed with REILD.

## Discussion

Based on the available evidence RE seems a promising addition to the currently applied downstaging and bridging strategies. The combination of the anti-tumoral effect and simultaneous hypertrophy induction of the non-embolized segments may have clear advantages over preoperative PVE or in situ splitting techniques in terms of tumor control and morbidity.

**Fig. 2:**
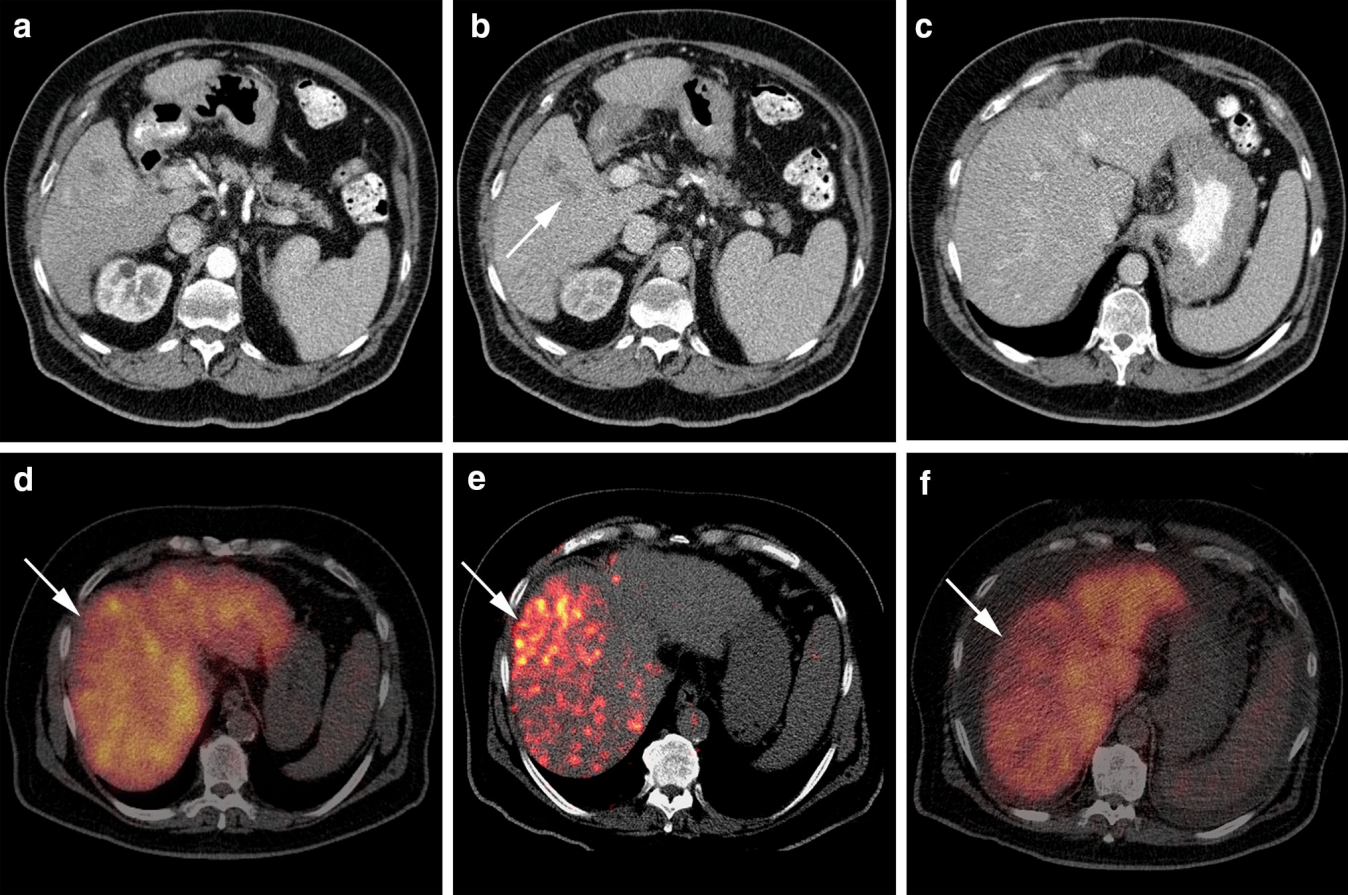
Decrease in ^99m^Tc-mebrofenin uptake after right lobar ^90^Y-RE treatment. **a** A solitary, hypervascular lesion is present in segment 5 with wash-out (*arrow*) on the later obtained portal venous phase (**b**), consistent with an HCC. **c** The liver has a cirrhotic appearance (note the nodular surface). No lesions are seen elsewhere in the liver. **d** Hepatobiliary scintigraphy before RE-treatment shows a fairly homogeneous uptake of ^99m^Tc-mebrofenin (cMUR: 3.0 %/min). **e** ^90^Y-PET/CT one day after right lobar treatment. ^90^Y has heterogeneously distributed in the right lobe with a higher dose in segment 4 and 8 (*arrow* in **d**, **e** and **f**). **f** Hepatobiliary scintigraphy 3 months after treatment. The uptake of ^99m^Tc-mebrofenin is decreased in segment 4 and 8, corresponding to the area of higher ^90^Y deposit on the ^90^Y-PET/CT

However, RE has an important downside. Radiation damage to the non-tumorous parenchyma will compromise the liver function (Fig. 2), with the inherent risk of REILD development and a decrease in regenerative capacity (as illustrated by the report of Bennink et al.) [61]. One of the most important risk factors for REILD is the absorbed dose or administered dose per target volume [80, 99]. Unfortunately, dose distribution in RE is non-uniform [100], thus difficult to predict, even if the tumor-to-non-tumor ratio is taken into account at activity calculation (partition model) [101]. In case of unilobar treatments with a sufficient FLR these uncertainties in dose–response relationships will be less important. However, in whole liver delivery (e.g. bilobar disease) the risk of REILD is higher [72, 80, 99].

Hypertrophy induction after RE is less pronounced and slower than after PVE [41]. On the other hand, the coinciding anti-tumoral effect of RE allows for more time for the FLR to hypertrophy. And if FLR hypertrophy is insufficient after RE, subsequent PVE/PVL can be considered [46, 62]. Another option might be combining transarterial and transportal RE. Toskich et al. recently reported on transportal RE of 2 HCC lesions (segment VII and VIII) in a patient not amendable for transarterial RE after repeated TACE, resulting in complete devascularization of the lesion in segment VIII [74].

Follow-up imaging plays a key role in determining the success of downstaging and the subsequent surgical planning. Furthermore, in TACE and RFA series tumor response prior to LTX seems to be associated with tumor recurrence [29, 31, 35, 102]. In the study by Tohme et al. histopathologic analysis showed complete tumor necrosis in 5/20 patients after RE, of whom 80 % had complete remission on imaging [57]. In contrary, Vouche et al. reported that only 50 % of patients with complete response (mRECIST) had complete tumor necrosis at histopathology (similar to previously reported TACE series [29, 32]) [75]. However, all explants showed 90–100 % necrosis after RE, with significantly more complete necrosis if the dose exceeded 190 Gy [75]. This discordance of pathology and imaging—regardless of the applied imaging criteria—illustrates the need for improvement, especially if TACE and RE are to be used in the curative setting.

In current practice MRI with hepatobiliary contrast agents (Gd-EOB-DTPA or Gd-BOPTA) is routinely used in the work-up for surgery and RE. In the future MRI with Gd-EOB might also be used to assess liver function [91]. Gd-EOB is processed by hepatocytes in the same way as ICG and ^99m^Tc mebrofenin [103]. Thus, the possible benefits of MRI with Gd-EOB as a liver-function test are obvious. MRI does not use ionizing radiation and has an excellent spatial resolution, resulting in an easier regional liver function assessment (compared to HBS) with the additional benefit of simultaneous assessment of the tumor status. A few studies reported decreased enhancement of irradiated segment(s) or lobes in the hepatobiliary phase (20 min after Gd-EOB-DTPA or 120 min after Gd-BOPTA injection) after external beam radiotherapy or brachytherapy [104, 105]. Seidensticker et al. correlated these findings with histopathology [105]. In 11/14 biopsies signs of radiation damage were present; all receiving >20 Gy and showing no enhancement 2 h after Gd-BOPTA injection. Another study assessed the reduction in enhancement in the hepatobiliary phase after RE [106]. After 60 days an evident reduction in enhancement was seen in the treated lobes with normalization of the enhancement after 4 months in most cases, suggestive of liver regeneration (i.e. a tolerable dose to the non-tumorous parenchyma). However, in some cases the enhancement of the treated lobes did not recover, indicative of permanent damage. These observations could be of value to estimate the regenerative capacity of treated lobes in case of repeated RE or post-RE surgery. Even though the parenchymal changes on MRI after RE are evident, the use of MRI with Gd-EOB-DTPA as a liver function test is not yet established and still requires further development and validation to be a clinically acceptable method [91, 107].

## Conclusion

The results of RE as a downstaging tool or bridge to LTX are encouraging. However, a better understanding of the dose–response relationships is imperative to prevent both insufficient tumor response and liver failure, especially in bilobar treatments and patients with a compromised liver function. An accurate measurement of the FLR function is essential to determine the feasibility of a safe resection (with HBS or in the future possibly with MRI).
